# Urinary Activin A: A Novel Biomarker for Human Acute Kidney Injury

**DOI:** 10.3390/diagnostics12030661

**Published:** 2022-03-09

**Authors:** Izumi Nagayama, Akito Maeshima, Daisuke Nagata

**Affiliations:** Division of Nephrology, Department of Internal Medicine, Jichi Medical University, Shimotsuke 329-0498, Japan; izumin@saitama-med.ac.jp (I.N.); nagatad@jichi.ac.jp (D.N.)

**Keywords:** activin A, acute kidney injury, urinary biomarker

## Abstract

Activin is a multifunctional cytokine belonging to the transforming growth factor (TGF)-β superfamily that regulates the growth and differentiation of cells in various organs. We previously reported that activin A, which is absent in normal kidneys, was significantly increased in the ischemic kidney, and that the blockade of activin action by follistatin, an activin antagonist, significantly enhanced tubular regeneration after renal ischemia, suggesting that activin A acts as an endogenous inhibitor of tubular repair after kidney injury in rodents. However, the role of activin A in human acute kidney injury (AKI) remains unclear. In this analysis, we measured serum and urinary activin A in human AKI (*n* = 39) and tested if activin A might serve as a biomarker for AKI. Urinary activin A, which was undetectable in healthy controls, was significantly increased in AKI (0.0 ± 0.0 vs. 173.4 ± 58.8 pg/mL, *p* < 0.05). The urinary activin A level in patients with AKI stage 3, was significantly higher than that in patients with AKI stages 1 and 2. Patients who required renal replacement therapy (RRT) had a significantly higher urinary activin A level than patients who did not require RRT. Urinary activin A might be a useful non-invasive biomarker for the severity of AKI.

## 1. Introduction

Acute kidney injury (AKI) represents a critical and potentially devastating condition in clinical settings, and has many complicated pathophysiological features, including tubular injury [1,2,3]. The incidence of AKI appears to be increasing globally. AKI is associated with morbidity and mortality, independent of multiple potential confounders. Serum creatinine is a simple and useful marker of renal function, but is unreliable during acute changes in kidney function, and does not accurately reflect kidney function until a steady-state has been reached.

Several molecules, such as urinary neutrophil gelatinase-associated lipocalin (NGAL) [4,5,6,7,8], interleukin (IL)-18 [9,10,11] and L-type fatty acid-binding protein (L-FABP) [12,13,14,15], have been identified as potential markers for the early detection of kidney damage, before serum creatinine increases. Kidney injury molecule-1 (KIM-1) has not only been proposed as a diagnostic biomarker, but also as a pro-recovery marker for AKI [16,17,18,19]. Recently, the combination of two cell-cycle arrest biomarkers, insulin growth factor binding protein 7 (IGFBP7) and tissue inhibitor of metalloproteinase 2 (TIMP-2), has been proposed to predict the onset of severe AKI with significantly greater accuracy than other biomarkers [20]. However, clinical biomarkers reflecting the pathophysiological phase of AKI are still lacking.

Activin A, one of the transforming growth-factor (TGF)-β superfamily, is an essential factor in renal organogenesis. Organ culture experiments showed that activin A inhibits branching morphogenesis of ureteric buds [21], as well as ureteric bud budding from the Wolffian duct [22]. In the kidney of transgenic mice overexpressing the truncated type II activin receptor, the number of glomeruli was significantly increased [23]. In an in vitro tubulogenesis model using Madin-Darby canine kidney (MDCK) cells, activin A was significantly inhibited, but, in contrast, follistatin (an antagonist of activin A) induced branching tubulogenesis [24]. These observations suggest that activin A negatively regulates branching morphogenesis during kidney organogenesis. Additionally, activin A is indispensable for the differentiation of metanephric mesenchyme [25]. We previously reported that activin A, which is absent in the normal kidney, appears in ischemic renal tubules in rats and inhibits tubular regeneration after renal ischemia [26]. Activin A regulates tubular cell growth and differentiation by modulating Pax-2, a transcription factor that is critical for kidney development [27]. In mice, urinary activin A was significantly increased after renal ischemia, but was undetectable in the urine of normal mice, and in a volume-depressed mouse model [28].

In the present study, we measured the levels of serum and urinary activin A in human AKI and found that urinary activin A was significantly increased in patients with AKI. Urinary activin A was correlated with severity and prognosis of AKI. A combination of urinary activin A, a useful non-invasive monitoring biomarker for the severity of AKI, and other biomarkers, might help us determine the appropriate timing for intervention.

## 2. Materials and Methods

### 2.1. Setting and Patients

The current analysis includes thirty-nine patients with AKI, treated at Jichi Medical University Hospital from December 2018 to February 2020. AKI was diagnosed and staged for severity according to Kidney Disease Improving Global Outcomes (KDIGO) guidelines [29]. Exclusion criteria included pre-existing renal insufficiency (estimated GFR < 45 mL/min/1.73 m^2^ before the onset of AKI) or ANCA-associated vasculitis. Patients with diabetes were also excluded, because circulating activin A correlates with reduced kidney function and kidney injury markers in patients with diabetes [30]. Serum and urine were obtained from living kidney donors before nephrectomy (*n* = 16), serving as healthy controls (HC). Written informed consent was obtained from each patient and control subject for study participation. This study was approved by the Ethics Committee on Human Research of Jichi Medical University (Approval number A18-081, A18-089). All experiments were performed in accordance with the relevant guidelines and regulations.

### 2.2. Sample and Data Collection

Urine and serum samples were collected from patients with AKI at the time of diagnosis and were stored at −80 °C until analysis. In some cases, urine and serum were sequentially collected until discharge. Clinical data at the time of diagnosis, including age, sex, urine and serum biochemical parameters, complete blood count, complications, medications, number of days receiving renal replacement therapy (RRT), survival, and cause of death, were extracted from the patient’s medical records.

### 2.3. ELISA

Urinary and serum activin A (DAC00B), urinary NGAL (DLCN20), and urinary KIM-1 (DKM100) were measured by the Quantikine^®^ ELISA kit (R&D systems, San Diego, CA, USA) according to the manufacturer’s instructions. All standards and samples were assayed in duplicate.

### 2.4. Statistical Analysis

GraphPad Prism 8 (GraphPad software, San Diego, CA, USA) was used for statistical analyses. For two-group comparisons, normally distributed data were analyzed by a two-sided *t*-test, and skewed data were analyzed by the Mann–Whitney test or Wilcoxon test. When comparing the means of more than two variables, data were analyzed using the Kruskal–Wallis test followed by Dunn’s multiple comparison test to adjust the probability. Correlation was analyzed using Spearman‘s rank correlation test coefficient. *p* < 0.05 was considered significant. Normality was assessed by the Shapiro–Wilk test.

## 3. Results

### 3.1. Baseline Characteristics of the Patients

The baseline characteristics of the patients and healthy controls enrolled in this study are shown in Table 1. Twenty-six patients with AKI were male and thirteen were female; their mean age was 60.2 ± 1.9 years (mean ± SE). There were no significant differences in the frequency of comorbidities between AKI patients and healthy controls. Blood urea nitrogen (BUN), serum Cr, white blood cell (WBC), and C-reactive protein (CRP) were significantly higher, while serum sodium (Na), estimated glomerular filtration rate (GFR), and hemoglobin, were significantly lower in AKI patients compared to healthy controls.

### 3.2. Significant Increase in Activin A in the Urine of Patients with AKI

**Figure 1:**
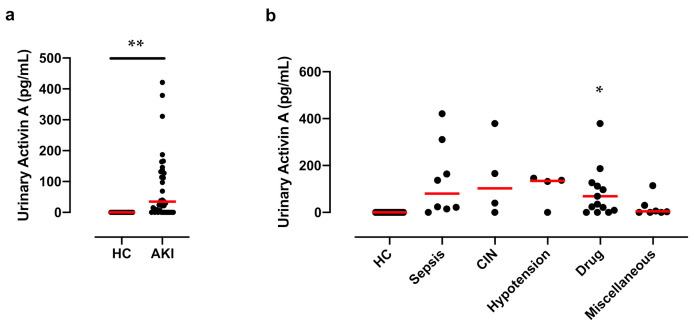
Urinary Activin A Levels in AKI Patients: (**a**) Urinary activin A levels in healthy controls (HC) (*n* = 16) and AKI patients (*n* = 39). ** *p* < 0.01. (**b**) Urinary activin A levels for each cause of AKI (sepsis, 8; CIN, 4; hypotension, 4; drug-induced, 15; Miscellaneous, 8). CIN, Contrast induced nephropathy. * *p* < 0.05 vs. HC. Data were analyzed by the Mann–Whitney test. Bar is the median value.

We first examined the concentration of activin A in the urine of healthy controls by ELISA and found that urinary activin A was undetectable in healthy controls. In contrast, urinary activin A was significantly increased in AKI patients (Figure 1a). Urinary activin A tends to be elevated in patients with AKI, independent of its cause. A significant increase in urinary activin A was observed in patients with Sepsis-associated AKI and drug-induced nephropathy compared to that in healthy controls (Figure 1b).

### 3.3. Urinary Activin A Is Associated with the Severity of AKI

**Figure 2:**
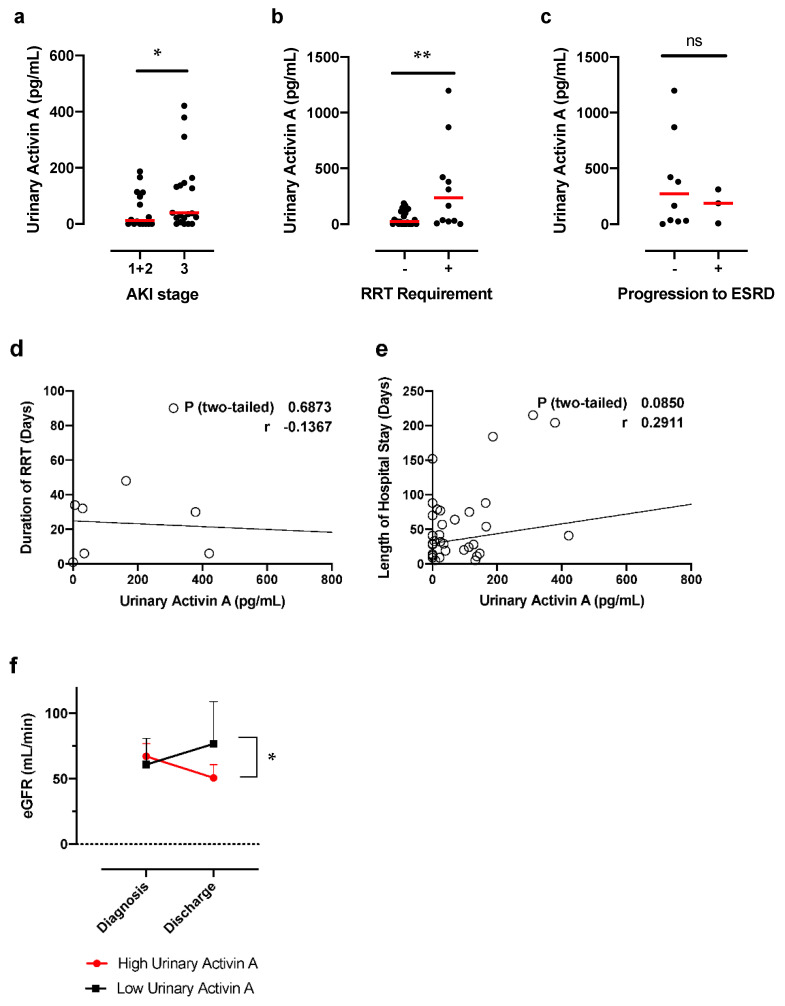
Urinary Activin A Levels and Renal Prognosis: (**a**) Urinary activin levels and severity of AKI according to KDIGO stage (stage 1–3). * *p* < 0.05. (**b**) Urinary activin A levels in AKI patients who did (+; *n* = 12) and did not (−; *n* = 27) require renal replacement therapy (RRT). ** *p* < 0.01. (**c**) Urinary activin A levels in AKI patients with (+; *n* = 5) and without (−; *n* = 14) progression to irreversible ESRD. N.S., not significant. (**d**) Correlations between urinary activin A and duration of RRT. N.S., not significant. (**e**) Correlations between urinary activin A and length of hospitalization. N.S., not significant. (**f**) Changes in estimated GFR after AKI in groups with high and low urinary activin A. * *p* < 0.05.

Next, we examined the relationship between urinary activin A and the severity of AKI. The number of patients in stage 1, 2, and 3 was 10, 6, and 23, respectively. Urinary activin A was significantly higher in patients with AKI stage 3 compared to patients with AKI stage 1 + 2 (Figure 2a). We also examined the difference in urinary activin A between the patients who required and did not require RRT. Urinary activin A in patients who required RRT was significantly higher than that in AKI patients who did not require RRT (Figure 2b).

On the other hand, urinary activin A levels did not significantly differ between patients who did and did not progress to irreversible ESRD (Figure 2c). Urinary activin A levels were not significantly associated with either duration of dialysis (Figure 2d) or duration of hospital stay (Figure 2e).

To further investigate if urinary activin A level at diagnosis of AKI is associated with renal prognosis, we compared estimated GFR at discharge between the patients with high and low urinary activin A. The two groups were sorted by median urinary activin A (76.1 pg/mL). There was no significant difference in estimated GFR at diagnosis of AKI between the two groups. In contrast, patients with high urinary activin A showed significantly lower estimated GFR at discharge compared to patients with low urinary activin A (Figure 2f).

### 3.4. Correlation between Urinary Activin A Level and Renal Function and Markers of Tubular Injury

**Figure 3:**
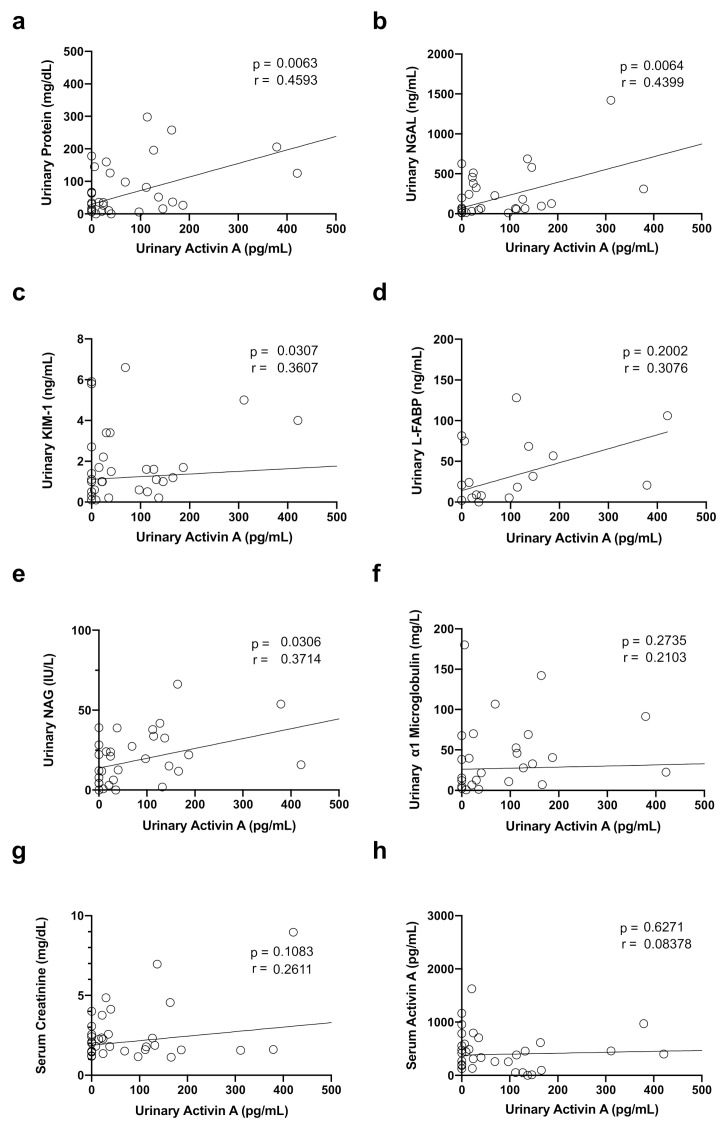
Correlation between Urinary Activin A levels and Renal Function and Markers of Tubular Injury: Correlations between urinary activin A and urinary protein level (**a**), NGAL (**b**), KIM-1 (**c**), L-FABP (**d**), NAG (**e**), alpha1 microglobulin (**f**), serum creatinine (**g**), and serum activin A (**h**), are shown.

We next examined the correlation between urinary activin A and several clinical parameters. Urinary activin A significantly correlated with urinary protein levels (Figure 3a) and urinary NGAL (Figure 3b), urinary KIM-1 (Figure 3c), and urinary NAG (Figure 3e), but not with L-FABP (Figure 3d), urinary alpha 1 microglobulin (Figure 3f), serum creatinine (Figure 3g), and serum activin A (Figure 3h).

### 3.5. Time Course of Changes in Urinary Activin A and Other AKI Biomarkers in a Patient with AKI

**Figure 4:**
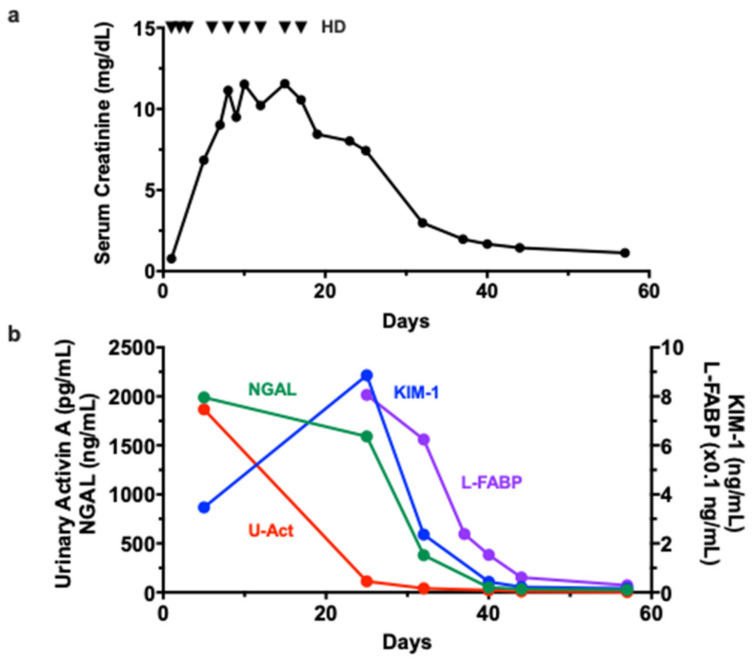
Time Course of Changes in Urinary Activin A and other AKI Biomarkers: Changes in serum creatinine (**a**) and AKI biomarkers (**b**) in a patient with drug-induced AKI. Triangle indicates the timing of hemodialysis (HD).

We finally compared the time course of changes in urinary activin A levels and other AKI biomarkers in one patient with drug-induced AKI. On admission, this patient showed oliguria and required RRT the day after admission. RRT was continued for 18 days, but urine volume gradually increased and renal dysfunction recovered thereafter (Figure 4a).

A high level of urinary NGAL was observed at diagnosis of AKI and persisted until 7 days after discontinuation of RRT. The elevation of urinary KIM-1 was also observed at diagnosis of AKI. Urinary KIM-1 peaked at 7 days after discontinuation of RRT, and decreased thereafter. Urinary activin A levels were significantly increased at the diagnosis of AKI, but, unlike NGAL and KIM-1, rapidly decreased before the normalization of serum creatinine (Figure 4b).

### 3.6. Urinary Activin A in Patients with Different Causes of AKI

**Figure 5:**
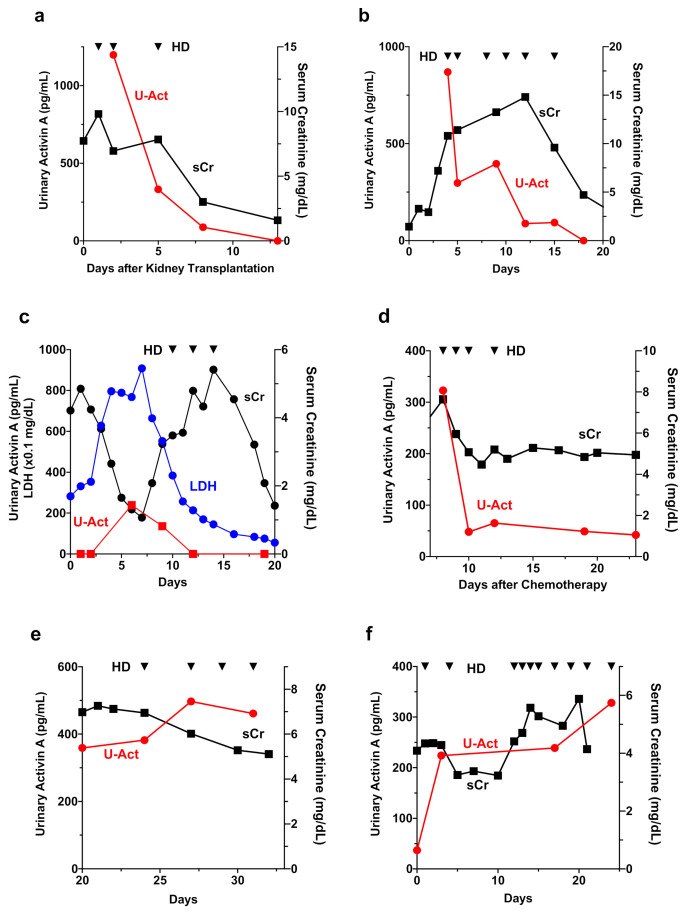
Time Course of Changes in Urinary Activin A in Patients with AKI. (**a**–**f**): Changes in serum creatinine and urinary activin A levels in patients with various causes of AKI, including cadaver-kidney transplantation (**a**), drug-induced nephropathy (**b**), Burkitt lymphoma accompanied by tumor lysis syndrome (**c**), chemotherapy (**d**), contrast-induced nephropathy (**e**), and cholesterol crystal embolism (**f**). Triangle indicates the timing of hemodialysis (HD).

We investigated the time course of changes in serum creatinine, urinary activin A and other urinary biomarkers in patients with different causes of AKI. In the case of cadaver-kidney transplant, urinary activin A levels were high immediately after renal transplantation, but gradually decreased and became undetectable thereafter (Figure 5a). In drug-induced AKI (Figure 5b) or AKI due to Burkitt lymphoma accompanied by tumor lysis syndrome (Figure 5c), urinary activin A levels returned to normal before the normalization of serum creatinine levels. In patients with chemotherapy-induced AKI requiring RRT, urinary activin A levels were increased immediately after the initiation of chemotherapy, but rapidly decreased thereafter (Figure 5d). In contrast, in AKI patients who progressed to irreversible ESRD due to contrast-induced nephropathy (Figure 5e) or cholesterol crystal embolism (Figure 5f), high levels of urinary activin A persisted until discharge.

## 4. Discussion

The source of urinary activin A in patients with AKI could not be elucidated in this study, because renal histological analysis is generally difficult in cases of AKI. Previous data from mice with renal ischemia raised the possibility that urinary activin A might be released by tubular cells after AKI. In normal mice kidneys, the beta-A subunit of mRNA for activin A, as well as activin A protein, are absent, although they became detectable in the cytoplasm of proximal tubular cells in ischemic kidneys. Furthermore, urinary activin A levels significantly correlated with activin A-positive areas in the kidney after renal ischemia [28]. In this study, we found no significant correlation between serum and urinary activin A levels in AKI patients, supporting the idea that urinary activin A is derived from the kidney, and not from the blood.

Urinary activin A was significantly higher in patients with AKI stage 3 compared to patients with AKI stage 1 + 2 (Figure 2a). Urinary activin A in patients who required RRT was significantly higher than that in patients who did not require RRT, suggesting that urinary activin A reflects the severity of AKI patients. To support this idea, previous data demonstrated that urinary activin A levels significantly correlated with the damaged area of the kidney after renal ischemia in mice [28]. A significant increase in urinary activin A was observed in AKI patients (Figure 1a) and urinary activin A level was associated with AKI stages (Figure 2a). There was a significant correlation between urinary activin A and urinary NGAL, an early diagnostic marker of AKI (Figure 3b). Therefore, it is also possible that urinary activin A is helpful for the early diagnosis of AKI. However, it is difficult to address this issue based on the results above, because urinary activin A was measured at the time of diagnosis of AKI and the timing of the sample collection after the onset of AKI was different in each patient in this study. To clarify this issue, a prospective study with patients at high risk of developing AKI should be planned.

Urinary activin A might be useful for monitoring the transition of AKI to CKD, in addition to its role as a biomarker for monitoring AKI status. Maladaptive repair after AKI has been noted as an important factor in CKD. Activin A not only suppresses tubular proliferation, but also promotes renal fibrosis [31]. Since activin A is a potent activator of renal interstitial fibroblasts [31], persistently high levels of urinary activin A might be a trigger leading to the transition of AKI to CKD. In this study, time course changes in renal function were observed in patients with various causes of AKI (Figure 5). In AKI patients who required but discontinued RRT until discharge (Figure 5a–d), urinary activin A tends to normalize before the discontinuation of RRT. In contrast, urinary activin A remained at high levels until discharge in AKI patients with progression to irreversible ESRD (Figure 5e,f). In a previous study, administration of recombinant follistatin, an endogenous activin antagonist, reduced the fibrotic area in rats with unilateral ureteral obstruction kidneys [32]. In another study, sotatercept, a ligand trap for the activin type IIA receptor, decreased renal fibrosis and proteinuria [33]. Taken together, neutralization of activin A by follistatin or other reagents might stop the transition of AKI to CKD in patients with persistently high levels of urinary activin A. Further study will be needed to address this issue.

Our study has several limitations. We analyzed a small number of subjects recruited from a single center, which may not be representative of the broad spectrum of AKI. Secondly, urinary activin A level at admission did not predict the progression of ESRD in this study, limiting its usefulness as a predictive tool. The prognostic value of urinary activin A in patients with less severe AKI is not guaranteed, because most patients in this study suffered from severe AKI.

## 5. Conclusions

In conclusion, urinary activin A was significantly increased in AKI patients and was associated with the severity and prognosis of AKI. The combination of urinary activin A and other biomarkers might help us monitor AKI status and determine the appropriate timing of interventions.

## Figures and Tables

**Table 1:** Baseline characteristics of the patients.

	AKI *n* = 39	Healthy Control *n* = 16	*p* Value
Age, years, mean ± S.E.	60.2 ± 1.9	54.9 ± 2.9	0.097
BMI, kg/m^2^, mean ± S.E.	24.2 ± 0.7	23.3 ± 0.7	0.711
Male gender, *n* (%)	26 (66.6)	2 (12.5)	0.058
Weight, kg, mean ± S.E.	63.9 ± 2.2	60.1 ± 3.2	0.383
Complications, *n* (%)			
Diabetes	0 (0)	1 (6.2)	0.089
Hypertension	25 (64.1)	7 (43.7)	0.059
Dyslipidemia	9 (23)	1 (6.2)	0.089
Old myocardial infarction	0 (0)	0 (0)	0.438
Old cerebral infarction	3 (7.6)	0 (0)	0.264
Angina pectoris	2 (5.1)	0 (0)	0.433
Chronic obstructive pulmonary disease	0 (0)	0 (0)	0.587
Liver disease	2 (5.1)	0 (0)	0.438
Hematological data, mean ± S.E.			
Na, mEq/L	138.3 ± 0.8	142 ± 0.4	0.007
K, mEq/L	4.5 ± 0.1	4.1 ± 0.1	0.069
Cl, mEq/L	104.2 ± 0.8	105.9 ± 0.6	0.138
BUN, mg/dL	47.9 ± 5.0	14 ± 0.8	<0.0001
Creatinine, mg/dL	3.64 ± 0.58	0.68 ± 0.04	0.001
eGFR, mL/min	23.5 ± 2.6	79.9 ± 3.6	<0.0001
Hemoglobin, g/dL	10.8 ± 0.4	13.2 ± 0.2	0.001
Platelets, ×10^4^/μL	16.6 ± 1.7	26.0 ± 1.6	0.004
WBC, ×10^3^/μL	10.2 ± 1.1	5.7 ± 0.4	0.021
CRP, mg/dL	8.0 ± 1.5	0.2 ± 0.1	0.001
Urinalysis, mean ± S.E.			
Urinary protein, mg/dL	166.6 ± 57.0	-	-
NAG, IU/L	28.9 ± 5.5	-	-
α1MG, mg/L	57.8 ± 12.8	-	-
β2MG, mg/mL	13.7 ± 4.6	-	-
NGAL, ng/mL	783.4 ± 218.1	-	-
KIM-1, ng/mL	2.4 ± 0.4	-	-
L-FABP, ng/mL	213.5 ± 147.1	-	-
FENa, %	4.7 ± 1.4	-	-
FEUrea, %	35.5 ± 3.0	-	-

Data were collected from healthy controls and patients with AKI at the initial visit. Abbreviations: AKI, acute kidney injury; BMI, body mass index; CKD, chronic kidney disease; eGFR, estimated glomerular filtration rate; WBC, white blood cell; CRP, C-reactive protein; NAG, N-acetyl-β-D-glucosaminidase; α1MG, α1microglobulin; β2MG, β2 microglobulin, KIM-1, kidney injury molecule-1; NGAL, neutrophil gelatinase-associated lipocalin; L-FABP, liver-type fatty acid-binding protein; FENa, fractional excretion of sodium; FEurea, fractional excretion of urea.

## Data Availability

The datasets used and analyzed in the current study are available from the corresponding author upon reasonable request.
